# After providing end of life care to relatives, what care options do family caregivers prefer for themselves?

**DOI:** 10.1371/journal.pone.0239423

**Published:** 2020-09-25

**Authors:** Jiska Cohen-Mansfield, Shai Brill

**Affiliations:** 1 Minerva Center for Interdisciplinary Study of End of Life, Tel-Aviv University, Tel Aviv, Israel; 2 Department of Health Promotion, School of Public Health, Sackler Faculty of Medicine, Tel-Aviv University, Tel Aviv, Israel; 3 The Herczeg Institute on Aging, Tel-Aviv University, Tel Aviv, Israel; 4 Minerva Center for Interdisciplinary Study of End of Life, Tel-Aviv University, Tel Aviv, Israel; 5 Beit Rivka Medical Center, Petah Tikva, Israel; 6 Tel-Aviv University, Tel-Aviv, Israel; University of Auckland, NEW ZEALAND

## Abstract

**Objectives:**

We examined how caregivers who had cared for a relative at end of life (EoL) wished to be cared for in the event that they experienced advanced dementia or physical disability in the future, and what factors influenced their preferences for EoL care.

**Methods:**

In this mixed-methods study, 83 participants, recruited from multiple sources in Israel, were interviewed concerning socio-demographic factors, health status, past experience with EoL, preference for extension of life vs. quality of life (QoL), willingness to be dependent on others, and preferences for EoL care.

**Results:**

In case of advanced dementia, 58% preferred euthanasia or suicide; around a third chose those for physical disability. Care by family members was the least desired form of care in the advanced dementia scenario, although more desirable than institutional care in the physical disability scenario. QoL was rated as the highest factor impacting preferences for EoL care. Men demonstrated a higher preference than women for extension of life over QoL.

**Conclusion:**

Our study points to the need for society to consider solutions to the request of participants to reject the type of EoL experienced by their relatives. Those solutions include investing in improving the quality of life at the end of life, and offering alternatives such as euthanasia, which a large proportion of our participants found ethically and medically appropriate within the current system of care in the event of severe physical disability, and more so in the event of advanced dementia.

## Introduction

The End of Life (EoL) period is becoming recognized as a distinct phase of human existence, giving rise to new social, medical, legal and existential concerns. EoL refers to the period of decline leading to the time of death [1]. Although it is widely known that this stage of life has grown longer and more expensive, patients, family caregivers and the health system often find themselves unprepared for EoL.

Literature exploring the EoL period has mostly focused on “the good death” [2], attempting to define “successful dying,” [3] or on the use of advance directives (instructions specifying preferences regarding medical decisions when one is unable to express them) to improve the EoL process [4]. Other issues that have been studied include incongruence of preferences for care among patients and family caregivers on issues such as preferred location of terminal care [5–8], where “terminal” suggests that this is the last type of care provided when a person’s condition has deteriorated. Lack of agreement about such care might lead proxies to decide on types of care that are not desired by the dying patients themselves. Related to this, many patients who prefer dying at home end up dying in institutions [6, 9].

EoL care preferences vary according to individual and cultural differences, as well as among various stakeholders in EoL care, i.e., patients, relatives and paid care-providers [10]. A review article [3] specified 11 attributes of good death including a dying process compliant with the patient’s advance directives, pain-free status and emotional well-being. Among the attributes, pain-free status, support by family, respect for patient, euthanasia in the case of unbearable suffering, and achieving a sense of completion were considered most important [2, 10].

Whereas some studies found that using advance directives can improve the EoL process for both patients and caregivers by reducing anxiety and depression [11], other studies reported that advance directives often fail to improve EoL decision making [12] or experience. Patients often state preferences in their advance directives based on incomplete medical information, or include text which is difficult to interpret later [13]. For example, patients can state that they do not wish intervention if their condition becomes”terminal,” but definitions of “terminal” are not uniform, thus rendering its determination subject to interpretation [14].

Identifying the attributes, or types of care desired by people at the last stage of EoL is essential for honoring people’s EoL care preferences and trying to close the substantial gap between the usual death (involving processes such as a prolonged disease process and care incompatible with patient preferences) experienced by most people, and the good death. For example, although most people desire a swift death [3], swift death was found to occur in only 7.1 percent of cases [15]. Moreover, a good death is often defined as involving patient control over care, such as the patient exercising control of location of care, timing of ADLs and use of life-sustaining treatments, whereas in reality there is often incomplete knowledge of patient care preferences and poor communication between medical staff and surrogates [13, 16, 17]. Additionally, despite most people reporting that they do not wish their EoL to be a burden to their family, a large majority of family caregivers (77%) reported that they lost time from work due to caregiving responsibilities [18], and that their needs for housekeeping services, caregiver respite, and in-home nursing care were not sufficiently met [19]. The emotional burden of EoL is substantial, not only for patients, but for family caregivers who often feel lonely, isolated and fearful [20].

Research on the factors people consider with respect to their EoL care preferences is limited. People's conceptions of the differences between the good and usual death may be affected by society’s general avoidance of thinking about death or by people's "death anxiety" [21–23]. Those who are technologically or medically oriented tend to view death as a lost fight against disease, rather than as a natural and inevitable part of life [24]. One study concerning life sustaining treatments identified the value of "quality of life" (QoL) vs. "extension of life" [25] as impacting preferences for such treatments. Another study emphasized the factors of fear of death or dying, and will to live [26]. Research on preferences for physician assisted suicide found the wish to avoid dependence as an important factor in such decisions [27]. In general, internal states, such as values and outlooks emerged as better predictors than background information. Studies of the general public regarding preferences for care under specific scenarios [28, 29] found that preferences were affected by previous personal experience, religiosity, and age, as well as by the anticipated scenario under which EoL decisions are to be made.

In the context of Israel, most older persons (96.5%) live in the community [30]. For those with severe functional decline, use of around-the-clock live-in migrant care workers is a common practice, and is sometimes partially supported by government funds [31]. In Israel, when relatives of older persons live nearby, such relatives are often involved either in direct care or in supervising the care provided by migrant workers [32]. Institutional long-term care in Israel is rarely used, with only 2.6% of those aged 65 and older residing at geriatric institutions [33].

Given the complexity of EoL decision-making, and because personal experience acquaints one with such complexity, we decided to examine these issues specifically within the context of those who cared for a relative throughout the EoL process. Research about family caregivers’ decision-making for their own long term care EoL preferences has not been explored with the exception of a small qualitative study [34]. Therefore, the aims of our study are (i) to characterize preferences for EoL care through the full range of potential care options as expressed by such family caregivers in the event of a poor, rather than good, EoL scenario, (ii) to determine the factors influencing their preferences, and (iii) to compare the factors which emerged from the statistical analysis with the perceived factors as reported by participants. Drawing on previous research [25–27], we hypothesize that three main factors impact persons' preferences for EoL care: persons' internal states, such as the value ascribed to QoL vs. extension of life, the will to live, and the personal attribute of tolerating living in a state of dependence.

## Method

### Participants

Participants were 83 relatives who served as the primary caregivers during their relative’s EoL period. The mean age of caregivers was 59.7 years, and 67.5 per cent were female. Most were children (72.0%) or spouses (14.6%) of the care-recipient at EoL. Most participants were born in Israel (67.5%), followed by Russia/Eastern Europe (18.1%), Western Europe/North America (7.2%) and Asia/Africa (3.6%). All were Jewish. Data were collected between March 2013 and March 2015.

### Research design and methods

This is a mixed method study, which included both close- and open-ended questions within one interview for each participant. The study involved a primary quantitative analysis that is further informed by a qualitative analysis that seeks to provide a deeper understanding of some of the concepts studied in the quantitative analysis from the participants' perspectives. The quantitative analysis established participants’ preferences for care options as well as relationships among the different factors and care preferences, whereas the qualitative analysis clarified the meanings of these findings, and added depth, reliability, and credibility.

The research was approved by the ethical committee of Tel Aviv University and by the Rabin Hospital (ethics registration number RMC-0318-12). Inclusion criteria were that participants (i) be the primary caregivers of a relative at EoL (ii) speak fluent Hebrew or English, and (iii) live within a manageable geographic distance (a radius of about 60km) from the interviewer.

**Fig 1:**
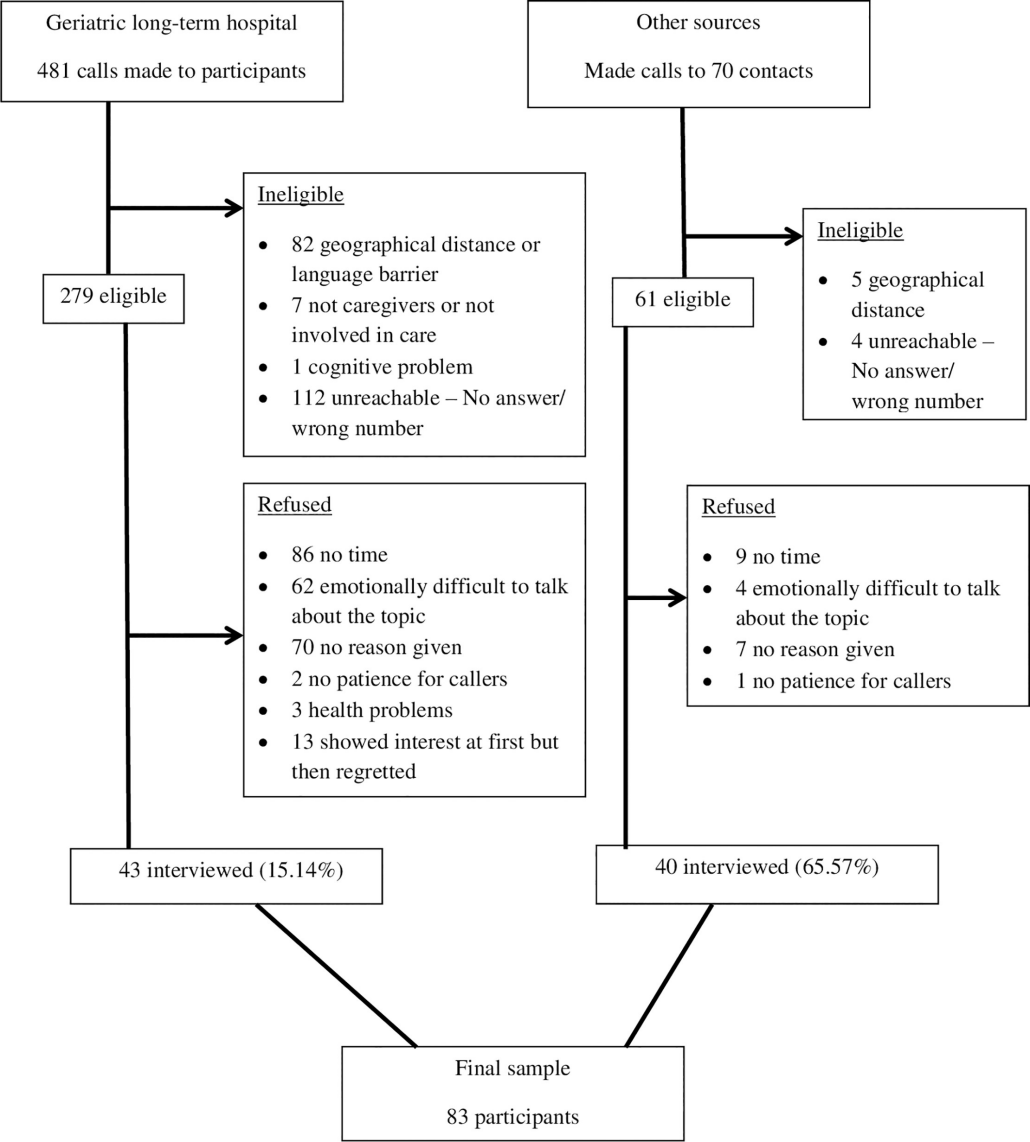
The process of participant recruitment.

Participants were recruited from among family caregivers who assisted relatives at a geriatric hospital, individuals involved in our research center’s discussion groups, referrals from the Alzheimer's Association, and via the snowball effect (see Fig 1). For those recruited from the geriatric hospital, letters were sent from the hospital to potential participants, i.e., relatives who had served or were serving as primary caregivers for relatives at EoL. The letters described the study, its goal, the involvement requested, and informed potential participants that a research assistant would contact them by telephone to request their consent to participate and to schedule an interview. Of the sample, 35% had cared for patients who had died a median of 1.5 years prior to the interview date, and 65% served as caregivers during the time of the study. During the recruitment process we verified that participants had taken care of a relative who died or who was at the end of life, and who had experienced advanced dementia or significant disability. Overall, about 75 percent of eligible participants did not consent to participate due to time constraints or emotional difficulty with the topic. For those who agreed to be interviewed, a meeting was scheduled at locations convenient to the participants (their home, the university, or another location). Informed written consent was obtained prior to the interview. The face-to-face structured interviews, including both the quantitative and qualitative questions, lasted two to three hours. At the end of the interview participants were given gift certificates with a value of about $25 in appreciation of their time and effort.

### Measurements

The interview consisted of the following sections:

### Background variables

1. ***Socio-demographic*.** Included participant age, sex, marital status, country of origin and level of religiosity. Religiosity was operationalized as a 4-point ordinal scale (1 = secular, and 4 = ultra-orthodox).
2. ***Health status*.** Included an item inquiring about the participant’s current health status on a 4-point ordinal scale (1 = bad, and 4 = excellent) based on the Medical Outcome Study [35].

#### Participant’s relationship to family and friends

was assessed via the following questions: “With how many family members or friends did you talk last week?”, “How many times did you talk with family members or friends last week?” and “How many people do you feel close enough to that they could take care of you if you were in need?”, (Cronbach’s Alpha = .755), which were collapsed to a new ‘Closeness to support network’ variable which was the mean of the three variables.

1. ***Past experience with EoL*.** This included the items, “To what extent did your relative suffer at EoL?” operationalized on a 6-point scale (1 = ‘Not at all’ to 6 = ‘Terrible suffering’), “Were your relative’s EoL needs fulfilled?” which was operationalized on a 5-point scale (1 = 'not at all' to 5 = 'completely') and the dichotomous item “Was there something in your relative’s EoL care that you would have wanted to be different?”
2. ***Willingness to be dependent on others*.** This included the item, “To what extent do you agree with the following statement: I can deal with being dependent on others for a long time” (measured from 1 = ‘totally do not agree’ to 7 = ‘totally agree’).

### Internal processes

1. ***The quality of life values inventory* [25].** This inventory assesses the level of agreement with five statements related to preference for extension of life versus QoL. Statements were rated on a 7-point Likert scale. Cronbach’s Alpha for the inventory was .881. The items’ mean yielded the overall ‘Preference for extension of life over quality of life’ score.
2. ***Will to live* [36]** was measured on a scale from 1 = no will to live, to 5 = very strong will to live.

#### Preferences for end of life care

The EoL care preferences section consisted of two undesirable EoL scenarios: (i) advanced stage dementia with loss of physical and mental capabilities, and (ii) loss of physical capabilities, such as paralysis, but with retention of cognitive function. The rationale for using these two scenarios was that they are the most common scenarios in which preferences for EoL long term care are decided in Israel and many other locations. For example, the Israel National Insurance Institute provides some support for home care or for live-in care by migrant worker based on ADL function and on need for dementia care and supervision [37–39]. For both questions, participants were given the following choices: (i) care in a nursing home/long term care institution, (ii) care by a caregiver at home, (iii) care by family at home, (iv) suicide, (v) euthanasia (euthanasia is currently illegal in Israel, however it is a generally known practice, and the topics of assisted suicide tourism [40] and euthanasia are part of public discussions [41]), and (vi) “I do not want to think about it.” For the bivariate analyses and path analysis, responses were further collapsed down to either 'Care' (responses (i), (ii) and (iii)) or ‘Termination of Life’ (responses (iv) and (v)). Response (vi) was marked as ‘missing data.’ All participants were asked about their reasoning for their choices under the different scenarios. These comments were transcribed during the interview and served as the basis for our qualitative analysis.

#### Perceived influences on EoL decisions

This section consisted of five questions assessing the extent to which participants believed each of the following variables impacted their EoL care preferences: (i) religion, (ii) financial situation, (iii) experience caring for a relative during EoL, (iv) burden on family, and (v) QoL. Each question was scored from (1 = not at all) to (5 = completely).

### Statistical analysis

Analyses were performed using IBM SPSS 23.0 and IBM Amos Graphics 23.0. McNemar’s test was used to assess differences between dichotomous EoL care preferences under the two EoL scenarios. We utilized Independent Samples T-tests, Chi-Squared tests and Pearson’s Correlations to assess baseline bivariate relationships between independent variables and dichotomous care preferences in the two EoL scenarios. A path analysis was computed using Structural Equation Modelling (SEM) to test the predictive power of background and internal process variables on EoL preferences. Only variables that most significantly correlated with the dependent variables in the bivariate analyses were entered into the model. The background variables of age, sex and ‘willingness to be dependent’ were entered as independent variables. The internal process variables of ‘will to live’ and ‘preference for extension of life over QoL’ were entered as mediators. Dichotomous care preferences in both the Advanced Dementia and Physical Disability EoL scenarios were entered as outcome variables. Missing values were generally few (e.g., none for age, sex, ability to handle disability, two for will to live etc.) However, nine persons were unable or unwilling to state a preference for the care they desired in a future scenario, and ten were unable to estimate the level of suffering of the relative for whom they provided EoL care. Missing data was accounted for by creating five imputations using SPSS. Pooled results from the five imputations were calculated using Rubin’s Rules [42].

### Qualitative analysis and integration of quantitative and qualitative data

In order to more fully understand the quantitative results, we conducted a thematic analysis of the comments participants provided when asked to elaborate on their choices. Thus, the qualitative analysis was integrated with the quantitative one by focusing on those responses which elucidated the meanings of the quantitative analysis. Thematic analysis is a method to systematically organize recurring patterns found in data into themes and sub-themes [43]. All data were transcribed and translated by research assistants with a degree in social sciences. Subsequently, the data were coded independently into themes by different members of the research staff and then refined during discussions which led to a consensus on a set of themes.

## Results

### End of life care preferences across scenarios

Care preferences varied between the two EoL scenarios (Advanced Dementia and Physical Disability). These results are summarized in Table 1. When presented with the scenario of Advanced Dementia, a majority (54.2%) of participants, preferred euthanasia, followed by ‘Care by (non-family) caregiver at home’ (23.6%), and ‘Care in a long-term care institution’ (13.9%). When presented with the alternative scenario involving Physical Disability at EoL, most participants (54.8%) chose ‘Care by a (non-family) caregiver at home,’ followed by ‘Euthanasia’ (27.4%), and ‘Care by family at home’ (8.2%). When comparing the dichotomous choice of receiving care versus ending life, a significant difference was found between the two EoL scenarios (p < .001) where participants were more likely to choose to receive care in the Physical Disability scenario than in the Advanced Dementia scenario.

**Table 1:** Care preferences in “not good” end of life scenarios.

EoL Preference	Dementia		Physical Disability	
	N	%	N	%
Nursing home/ Long Term Care Institution	10	13.7	4	5.5
Caregiver at home	17	23.3	40	54.8
Family care at home	3	4.1	6	8.2
Suicide	3	4.1	3	4.1
Euthanasia	40	54.8	20	27.4
I do not want to think about it		5	2	
Did not answer		5	8	

### Self-attributions of reasons for care preferences

Participants attributed ‘quality of life’ (4.24 out of 5) and ‘burden on family’ (3.95) as factors that had the largest impact on their EoL care preferences. The factor with the smallest average perceived impact on preferences was ‘religion’ (1.38) (see Table 2). In order to better understand these findings, we compared ratings for those describing themselves as religious (n = 17) to those describing themselves as secular (n = 57), and found that those who reported not being religious were more likely to perceive that religion had no effect on their EoL care preferences (χ^2^ = 13.72, df = 1, p < .001) compared to those who reported being religious.

**Table 2:** Self-attributions of reasons for EoL care preferences.

Influence	Mean perceived impact on EoL preferences (out of 5)
Religion	1.38
Lack of money	1.78
Experience with relative at EoL	3.53
Burden on family	3.95
Quality of Life	4.24

Scale: 1 = did not influence at all to 5 = completely influenced

### Predictors of EoL care preferences

#### Bivariate analyses between background and internal variables and EoL care preferences

*Advanced dementia scenario*. We conducted bivariate analyses to determine predictors of EoL preferences in the choice between receiving care or preferring death in the Advanced Dementia scenario. Those who preferred to receive care were found to be more religious (t(37) = 2.67, p = .011) and had a higher level of willingness to be dependent (t(53) = 3.018, p = .004) than those who preferred to terminate their life. Additionally, those who preferred to receive care had a greater will to live (t(63) = 3.585, p = .001), and placed more value on extension of life than on QoL (t(47) = 3.39, p = .001) compared to those who chose to ‘terminate life.’ Age, sex, current health, marital status, country of origin, relationship to family, and the experience of caring for a relative at EoL were not significantly related to EoL care preferences.

*Physical disability scenario*. Bivariate analyses revealed that, similar to the Advanced Dementia scenario, when faced with Physical Disability at EoL, those who opted to receive care had a significantly higher level of religiosity (t(70) = 3.345, p = 0.001) and were more willing to be dependent on others (t(57) = 2.807, p = .007) than those who preferred to terminate their life. Additionally, those who chose to receive care reported a higher will to live (t(71) = 2.268, p = .02), and were more likely to value extension of life over QoL (t(68) = 3.590, p = .001). Age, sex, current health, marital status, country of origin, relationship to family, and the experience of caring for a relative at EoL were not significantly related to EoL preferences.

#### SEM path analysis

Dichotomous EoL care preferences in both the Advanced Dementia and Physical Disability scenarios were entered as dependent variables. Although age and sex were not significantly correlated with either outcome variable, they were included as controls. Based on bivariate analyses, the independent variables ‘willingness to be dependent,’ ‘will to live’ and ‘preference for extension of life over QoL’ were included in the path analysis.

The model fit summary yielded the following results: χ^2^(2) = .669; p = .716, χ^2^/df = .335. Further model fit indices confirmed that the overall model showed a good fit to the data sample as Normed Fit Index (NFI) = .994, where a value greater than .95 is considered ‘good’ [44], Comparative Fit Index (CFI) = 1.000 which similarly indicates a goodness-of-fit [45] and Root Mean Square Error of Approximation (RMSEA) = .000 which represents a model of ‘excellent’ fit [46].

**Fig 2:**
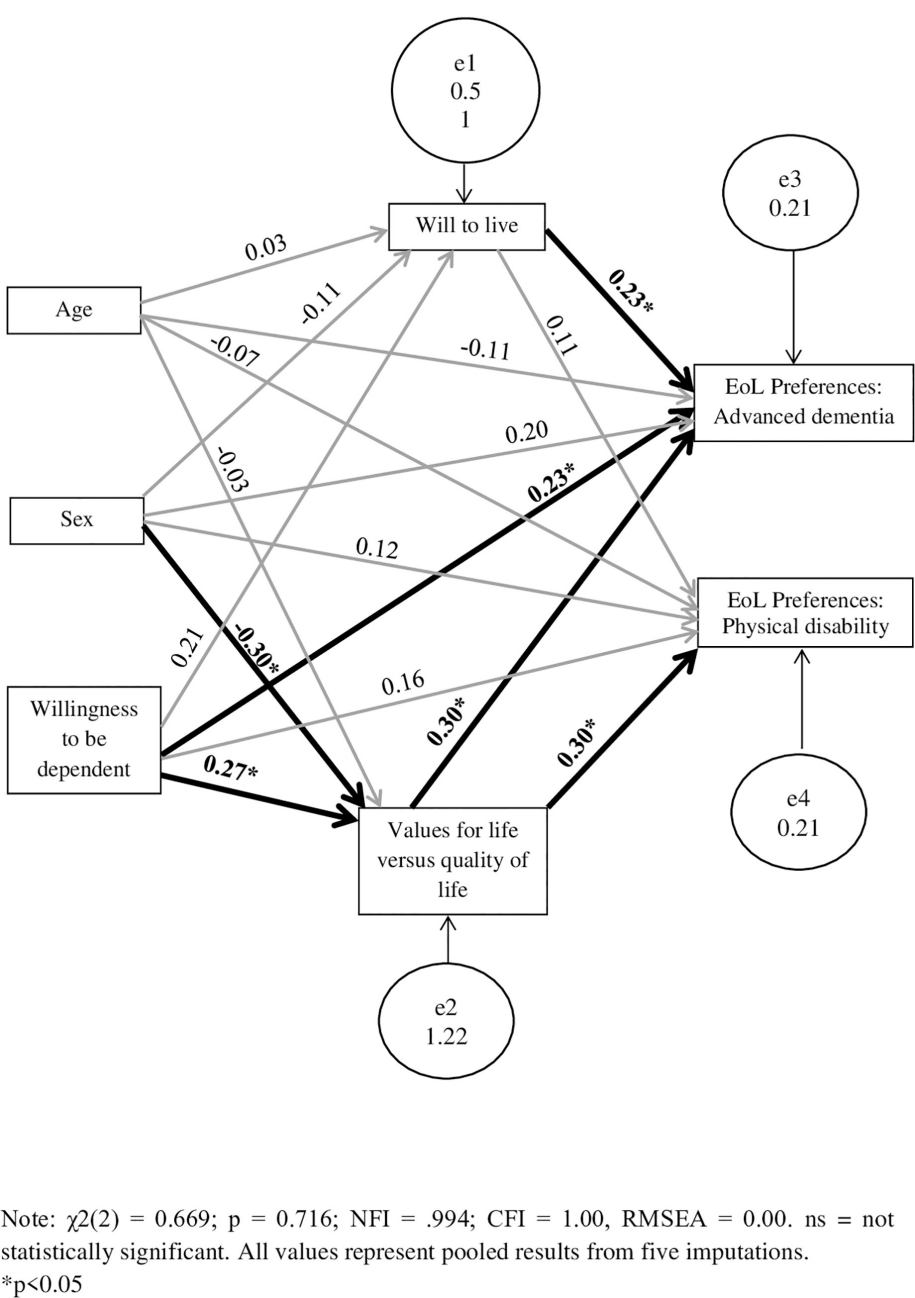
Structural Equation Modelling displaying the relationships between background variables, internal processes and care preferences for EoL care.

*Direct effects*. The full SEM model is displayed in Fig 2. Beta coefficients and p-values for the direct effects are available from the authors. Age did not have any significant direct effects on either the mediators or the outcome variables. Sex had a significant direct effect on ‘Preference for extension of life over QoL’ (β = -0.30, p<0.01) and ‘Willingness to be dependent’ had a significant direct effect on both ‘preference for extension of life over QoL’ (β = 0.27, p = 0.01) and on the dependent variable, ‘EoL Preference: Advanced Dementia scenario’ (β = 0.23, p = 0.04). Finally, both ‘will to live’ (β = 0.23, p = 0.02) and ‘preference for extension of life over QoL’ (β = 0.30, p = 0.007) were found to have significant direct effects on care preferences in the Advanced Dementia scenario, whereas only ‘preference for extension of life over QoL’ significantly predicted (β = 0.30, p = 0.01) EoL care preferences in the Physical Disability scenario.

*Indirect effects*. Age did not have any significant indirect effects on the outcome variables. Similarly, sex was not found to have a significant indirect effect on EoL care preferences either in the Advanced Dementia scenario (β = -.16, p = 0.058) or in the Physical Disability scenario (β = -0.10, p = 0.06), despite approaching significance. ‘Willingness to be dependent’ was found to have a significant indirect effect on EoL care preferences in the Advanced Dementia scenario (β = 0.13, p = 0.03). This indicates that the variable ‘preference for extension of life over QoL’ serves as a partial mediator for the effects of ‘willingness to be dependent’ on ‘EoL care preferences in the Advanced Dementia scenario.’ Additionally, the indirect effect of ‘willingness to be dependent’ on ‘EoL preference in the Physical Disability scenario’ approached significance (β = 0.10, p = 0.06).

### Qualitative analysis

The qualitative analysis focused on narratives that illuminated the meaning of the quantitative analysis, thus highlighting such issues as why so few participants chose family care, what meaning was attributed to home care or care by family in this context, and why so many participants chose death over life, etc. as denoted in the headings below. The quotations in the following text contain the participant number (#), gender, age and their care preference in the scenario of Advanced Dementia (d) and/or Physical Disability (pd).

#### Quality of life as a leading determinant for the choice of death

The wish to maintain an acceptable QoL pertained to avoidance of suffering and the wish to terminate life when it is no longer enjoyable for the person or family: “If there is no quality of life, and there is no hope for quality of life and enjoyment neither for me nor for the family, why extend it?" (#17-M-Age 68-euthanasia (d, pd)).

Some emphasized the importance of ending life in dignity, in the sense of dying as an independent person or in the way in which one wanted to be remembered by others: “I want to control the way I leave the world, finishing my life. I want to die with dignity, [as] an independent person.” (#21-F-Age 73-euthanasia (d) or suicide (pd)); “I do not want them [relatives] to remember me in this way, wretched and helpless.” (#8-F-43-euthanasia (d)).

Many participants considered the Advanced Dementia scenario as a life not worth living: “A physically handicapped person is a part of life; a mentally handicapped person is a walking dead.” (#26-M-32-nursing home (d), caregiver (pd)).

In some of these cases, euthanasia was considered preferable when one reached a state with very limited vitality: “… People want to live… But once it's over and there is no more than ten per cent vitality in life, then one has to end it.” (#50-M-51, euthanasia (d, pd)). Others mentioned that they preferred euthanasia when the migrant caregiver could no longer care for them, presumably due to their deterioration of function.

#### Participants’ ethical concerns when deliberating for or against ending life

While many participants opted for euthanasia or suicide, others raised ethical or religious concerns: “I do not think of it [euthanasia] as an option, because of my religious beliefs. I see it as a form of suicide.” (#57-M-48, caregiver (d, pd)). Others referred to the value of life: “Life has to be extended at any price for even another small moment.” (#9-F-41-nursing home (d), caregiver (pd)). One participant emphasized that even a life with Advanced Dementia is still worth living: “A person with advanced dementia is an active person […] a person with dementia has a world of his own and does not deserve euthanasia.” (#62-F-65-nursing-home (d, pd)).

#### Participants’ awareness and knowledge about the practicalities of euthanasia

The choice of care was also influenced by financial or legal considerations: “It is quite complicated, it is illegal [euthanasia]. I'll find a way to end my life myself, I have a lot of determination to do this, long ago I thought about it." (#21-F-73, euthanasia (d), suicide (pd)). Others indicated concerns regarding committing suicide: “Suicide—I never thought [about it], maybe I'm not heroic enough for this.” (#53-M-92, euthanasia (d, pd)); or that it is not feasible for someone with dementia, “Someone with dementia cannot do it [suicide], I will not make the connection between the pill box and suicide, it's not realistic.” (#21-F-73, euthanasia (d) or suicide (pd)).

Several participants referred to the difficulty of planning ahead, stating that their current choice might change depending on future circumstances. One participant framed this aptly: “[…] There is a saying: 'Man plans and God changes'…. I know I'm not in control […] I may think that I plan, but in the end, I am on a pathway that someone else arranged for me.” (#40-F-58-suicide (d), nursing-home (pd)).

#### Avoiding becoming a burden as an underlying factor for participant choice of care

Many participants mentioned that they did not want to be a burden on their families: “I do not want to be a burden on the family. Do not want [them] to stop [their] life because of me.” (#28-F- 51-caregiver (d, pd)).

Even when a relative is willing to provide caregiving or if a migrant caregiver is employed, it can take a toll on other family members: “I knew someone who took care of her husband for ten years at home. She thought she was doing holy work, but it harmed the children and the grandchildren.” (#46-F-66-caregiver (d, pd)).

Burden was noted as an issue even when a migrant caregiver could be provided: “What about my unfortunate daughters? They should run after the [migrant] caregiver to assure that she does not abuse me?” (#92-F-45-euthanasia (d, pd)).

The decision to avoid becoming a burden was sometimes related to a determination that life was only worth living as long as one was able to retain control and remain independent: “I'm not willing to reach a point where I cannot control my own affairs. I want to control [my life]; I do not want others to decide for me.” (#69-F-78-euthanasia (d, pd)).

#### Mixed reasoning concerning quality of care in different settings as an underlying factor for choice of care

The perception of the venue in which care would be optimal influenced the choice of caregiver: “I would prefer a personal caregiver, a person of trust who is professional, who receives money. […] It's much easier to get help from someone who works in it [professionally].” (#8-F-43-euthanasia (d), deliberating between options of care (pd)).

Some preferred institutionalized care: “It is better to be treated in a place that specializes in such care, assisted living or a nursing home." (#56-M-57-nursing-home (d), caregiver (pd)).

Others thought an institution would provide inferior care: “The institution–there is no respect for a person there. It is a kind of factory." (#18-M-51-caregiver (d, pd)), or that they might be abused there (e.g., #36-F-65-caregiver (d, pd)).

Some raised concerns about the possible lack of shared language and culture, and concerning insufficient professional experience of migrant workers: “People [who are] dependent on migrant workers have the problem of [a different] language and culture, also lack of experience of care for older persons.” (#59-F-49-caregiver (pd)).

#### Preference for closeness to loved ones and home as an underlying factor for choice of care

Some participants stated that they preferred to stay in their home with their family or a partner: “Home is important, it seems to me the [best choice]. [Staying] as much as possible in your environment.” (#11-M-61-caregiver). Yet, this raised the dilemma between being at home with family vs. not being a burden: “A conflict between my desire to be with everyone and maintaining their comfort.” (#9-F-41-nursing home (d), caregiver (pd)).

#### Personal experience as an underlying factor for choice of care

Throughout the interviews, participants demonstrated that their decisions were informed by their personal experiences of caring for family members, experiences shared by acquaintances, or observations of professionals involved with EoL care:

I would not want to leave home, to [enter] an institution…in this situation people are helpless–it is a tragedy for the person. We did everything in order not to get my parents out of the house, and my brother decided that…, he never forgave himself… –for the decision to finally take father out of the house. (#50-M-51, euthanasia (d, pd)).

## Discussion

This study is innovative in investigating care preferences under difficult EOL scenarios of individuals who served as primary caregivers for a relative at EoL, and by examining the factors which influenced those preferences–both via a statistical analysis and through their perceptions of influences. We used three methodologies to clarify the choice of care modality: a regression model using SEM, a direct question concerning the perception of the main factors affecting care preferences, and a qualitative analysis of participants’ interview narratives.

A notable majority, (58%) of our sample, preferred termination of life (euthanasia or suicide) in the Advanced Dementia scenario, with around one-third expressing the same preference in the Physical Disability scenario. A substantial proportion of people who experienced the EoL process as a caregiver for a relative found cognitive and physical debilitation to be worse than death [47, 48]. The higher rate of preference for care over termination of life in the Physical Disability scenario suggests that not all “less than ideal” scenarios are regarded equally. Consideration of the type of EoL scenario as an important determinant of participants’ choices is often missing from the literature on preferences for EoL care. While Advanced Dementia was mostly perceived as a “life not worth living,” Physical Disability was associated with greater control over the environment and potentially more manageable with the help of caregivers and technology. Thus, Advanced Dementia was perceived to be associated with a lower quality of life than Physical Disability, which is consistent with the literature that reports that mental disabilities, such as depression, are perceived as having a higher negative impact on daily functioning than physical disabilities [49]. This is also consistent with findings by Dassel, Utz [29], in which participants’ preference to remain alive and at home was lower in case of Alzheimer’s disease than in the case of severe physical disability, and by Clarke, Fistein [28] in which participants’ preference to bring life to a peaceful conclusion increased as dementia advanced.

The anticipated level of QoL emerged as a major determinant of preferences for EoL care, impacting the preferences both according to the regression model and according to self-reports. Participants defined QoL as retaining the ability to enjoy life–for themselves and for their relatives, and the opportunity to conclude life in dignity. Dying in dignity was often associated with a preference for euthanasia and suicide because it allows patients to determine how their life will end. In our study, the value placed on QoL informed participants’ preferences for continued care vs. death. Death, either through euthanasia or suicide, was more likely to be preferred in the Advanced Dementia scenario than in cases of Physical Disability, presumably because of the lower QoL associated with dementia. This finding raises important questions about current policies regarding euthanasia in Israel and beyond since most assisted death legislation currently requires that participants be terminally ill and also mentally competent. While there are some efforts to change physician-assisted death laws to include this option for people with mental disorders [50], even where physician-assistance death is legal, it is generally not available for persons with dementia. The question of whether euthanasia should be available to persons with advanced dementia, either directly or through advance directives, remains subject to debate and deliberation as to ethics and practicality [51, 52]. Thus, euthanasia is not presently a legally available option in the very circumstances in which our study’s participants perceived it to be most needed.

Of participants who preferred termination of life over receiving care, most desired euthanasia, despite acknowledging its illegality in Israel. The endorsement of euthanasia among terminally ill patients and family EoL caregivers has been confirmed in other studies [34, 53, 54]. The choice of euthanasia was guided by multiple influences, most notably the perception that in a state of dementia, one may be incapable of suicide, and that euthanasia might be experienced as an easier “good death” in contrast to the usual death, as suggested in Meier, Gallegos [3], because it is quick, painless, and controlled. A study by Emanuel, Fairclough [53], however, reported that only a minority of those who support euthanasia seriously consider this option for themselves. Yet patients think that it may offer a relatively good death, and that its availability as an EoL care option offers them greater peace of mind. As seen in our results, this topic remains highly controversial, and the evolving meaning of dignity and wellbeing of people who experience progressively more severe manifestations of physical and cognitive decline needs to play a part in future public deliberations, e.g., [55].

Quality of care and level of professionalism in different modes of care were sources of concern which impacted preferences for specific types of care. Those who preferred care at home were often conflicted because of their wish not to burden relatives. Similarly, perceived caregiver burden has been found to influence patients’ preferences for a palliative care unit or nursing home over being cared for at home [56, 57]. The wish to avoid becoming a burden was sometimes described in the context of desiring to maintain control and independence, which, in turn, was related to family dynamics and financial considerations. The experience gained from having cared for another person might also influence the fear of burdening others in the future [58].

Those who did not choose to die were most likely to choose to be cared for by a paid caregiver at home, the most common setting in which care is provided to frail older persons in Israel [59]. While care by family members was the least desired form of assistance in the Advanced Dementia scenario, it was held more desirable than institutional care in the Physical Disability case, perhaps because of a perceived lower caregiving burden associated with Physical Disability as compared to Advanced Dementia.

Compared to the other factors, religion was the least frequently cited factor affecting preferences for EoL care, but our sample consisted mostly of secular individuals. In contrast, multiple studies reported that religiosity affected EoL preferences, albeit in other populations [29, 60–62]. Since participants who identified as religious differed from those who identified as secular, a separate study with a larger representation of religious participants is required for understanding the effect of religion on EoL care preferences.

Gender emerged as influencing care preferences. For example, preference for values supporting extension of life over QoL was stronger in males, corroborating research by Arber, Vandrevala [63] who found that older women were twice as likely as men to oppose life-extending technologies. Similarly, older women were found to have a lower commitment to life than older men [36].

Preferences for future EoL care and death should be treated with caution. It has been argued that preferences expressed earlier in life often change when death becomes more imminent [64, 65], a time when patients might prefer to be relocated to hospitals and hospices to relieve family burden. This has important consequences for advanced care direction to enable maximum self-determination among patients and their caregivers, and for the development of services that accommodate those wishes [66].

The present study contributes to existing research in multiple ways. First, unlike other studies which generally examined only one or two options for EoL care, such as euthanasia or home care, this study presented the full range of care options available at EoL. The study also examined the topic from both quantitative and qualitative points of view, thus providing an understanding of factors that emerged as statistically significant, including the participants’ decisional deliberations. It thus clarifies not only the choice of preference, but also the factors which may inform and influence those preferences. The study is innovative by utilizing a sample that is intimately acquainted with the EoL experience through caring for a relative at EoL.

### Study limitations and future research

While the results confirmed our hypothesis about a relationship between EoL care preferences and internal states, the hypothesis concerning the impact of level of suffering observed in prior EoL experiences was not confirmed in the quantitative analysis. However, this aspect of the theory did emerge in the qualitative results, confirming studies that reported that past experiences with EoL care exerted an influence on whether participants undertook certain actions regarding their own EoL planning [34, 67]. The impact of past experience may require a more in-depth exploration of the various types of EoL experiences for caregivers, and the intensity level of those experiences; it would benefit from examininga greater range of experiences, since our sample included only caregivers with substantial EOL experience. Future research should also include those without experience in caring for a relative at EoL. Additionally, further study should compare a sample of those with relatively positive experiences with a sample whose experiences were unfavorable.

Another limitation in these analyses is the relatively small sample size. While about 75 percent of eligible participants chose not to participate due to time constraints or emotional difficulty with the topic, we obtained a sample of individuals willing to think about a subject of considerable personal weightiness, and willing to discuss their care preferences and reasoning. The very high rate of decline to participate may affect the generalization of the findings. Indeed, there is evidence, albeit from other samples and different research questions, that voluntary participation is subject to sampling bias, especially when the research topic is controversial [68]. This seemed to be the case in our study in regard to the variable of willingness to think about, and potentially make decisions about EoL care for oneself. This topic is likely to be a particularly sensitive one for those who recently lost a relative. Indeed, many of those who declined to participate expressed this very reason for declining.

As a general matter, a significant segment of the population is not prepared to tackle EoL care issues, as exemplified by the low rates of execution of advance directives [69]. Indeed, Haesen and Shaw [70] reported reasons for not executing advance directives which are similar to the ones given in our study by those who declined to participate. In this sense, the high prevalence of either denial concerning future death, or inability to confront one’s mortality may pose impediments to improving EoL care, and may warrant a separate investigation as to the meaning and implications of such feelings. While the opinions of those who responded to our questionnaire may not represent the whole population, the absence of opinion, or the unwillingness to confront these issues by the majority of the sample, may indeed represent a majority of the population.

Although the analyses showed a very good fit of the model obtained, our sample size resulted in inadequate power to detect less potent relationships, and thus some of the relationships which were not statistically significant in our model may prove to be significant when studied in a larger sample. While we could not determine whether participants' care preferences change when actually confronting various scenarios, the lack of relationship between the participants’ ages and their responses in this study suggests that temporal remoteness from the scenario does not affect preferences for EoL care.

## Conclusions

Our results suggest that people who cared for relatives at EoL consider multiple inter-related factors based on their past experiences of caring for relatives at EoL, and their values and inclinations when contemplating their own preferences for EoL care. A large portion of EoL caregivers was unwilling or unable to confront the gap they perceived between a good EoL and the usual EoL, but those who agreed to participate in our study were clear that the present EoL care system is unacceptable to them.

As the rate of suicide among older persons rises [71], and assisted suicide becomes legal in more places, questions are raised as to whether society is willing to invest in improving QoL at EoL, and whether it is willing to provide alternatives. The option of euthanasia may be psychologically helpful, and most of our study’s participants considered its availability to be ethical and medically appropriate within the current system of care. Our study points to the need to include these issues in public discourse about the living and dying experiences of patients and their caregivers at the end of life.
